# Atrioventricular Block: A Heralding Sign of Cardiac Allograft Rejection

**Published:** 2020

**Authors:** S. Shafaghi, F. Naghashzadeh, B. Sharif Kashani, N. Behzadnia, Z. H. Ahmadi

**Affiliations:** 1 *Lung Transplant Research Center, National Research Institute of Tuberculosis and Lung Diseases (NRITLD), Shahid Beheshti University of Medical Sciences, Tehran, Iran *; 2 *Tobacco Prevention and Control Research Center, National Research Institute of Tuberculosis and Lung Diseases (NRITLD), Shahid Beheshti University of Medical Sciences, Tehran, Iran*

**Keywords:** Heart transplant, Rejection, Second degree AV block, Arrhythmia

## Abstract

Heart transplantation is the treatment of choice for those with end-stage heart failure. However, despite improvements in immunosuppressive treatment, patients are at significant risk of allograft rejection, especially early after transplantation. Any changes in patient’s heart condition including reduced left ventricular ejection fraction, arrhythmia and any types of blocks need attention. Herein we report on a 29-year-old man who underwent heart transplantation 5 years before due to dilated cardiomyopathy. He was on immunosuppressive therapy and was good until one week before his admission, when he felt palpitation. Electrocardiography during palpitation showed a second-degree AV-block with heart rate of 60 beats/min. Echocardiography showed good left ventricular systolic function with no regional wall motion abnormality. The patient referred for coronary angiography and endomyocardial biopsy. The angiography was normal. The biopsy showed rejection compatible with ISHLT grade 2R. After treating the patient with 1.5 g methylprednisolone, the symptoms relieved and the block resolved. Bradycardia and second-degree AV-block late after heart transplantation could be a sign of cardiac allograft rejection and need more evaluation, especially endomyocardial biopsy.

## INTRODUCTION

Heart transplant is the standard treatment for those with end-stage heart failure. Cardiac allograft rejection is the most important complication post-transplantation. Acute cellular rejection (ACR) is the most common event during 6 months after heart transplantation. However, the incidence decreases by the time so that it decreases to 20%–30% in the first post-transplantation year [1]. Initially, the symptoms of rejection may be nonspecific including increased intracardiac filling pressure, and complaining of congestive symptoms. Palpitation, or less commonly syncope, may result from arrhythmias triggered by myocardial inflammation. Less commonly rejection can also be associated with bradyarrhythmia and atrioventricular (AV) block [2].

AV block (mostly complete) has been reported late after heart transplantation. It has several etiologies including post-operative injury, progressive conduction system disease associated with coronary artery disease, left ventricular (LV) dysfunction, acute and chronic rejection, and injury from endomyocardial biopsies [2].

## CASE PRESENTATION

**Figure 1:**
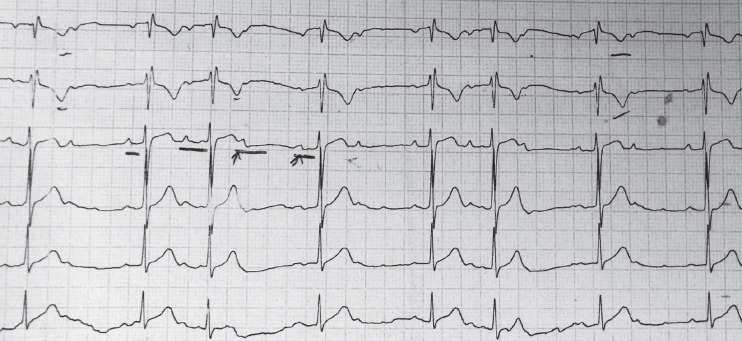
ECG before treatment demonstrating second-degree AV-block

A 29-year-old man with history of dilated cardiomyopathy underwent orthotopic heart transplantation 5 years before. He was on immunosuppressive therapy (cyclosporine, prednisolone, and mycophenolate mofetil). Two days before his admission, he developed palpitation and referred to a local clinic. ECG showed a second-degree AV-block; the heart rate was 60 beats/min (Fig 1).

**Figure 2:**
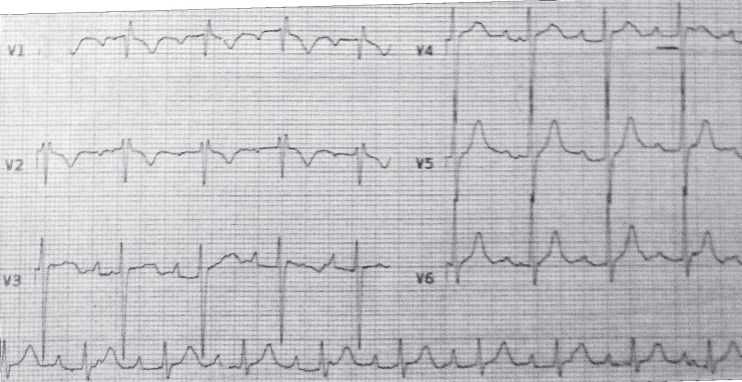
ECG one day after treatment showing first-degree AV-block

**Figure 3:**
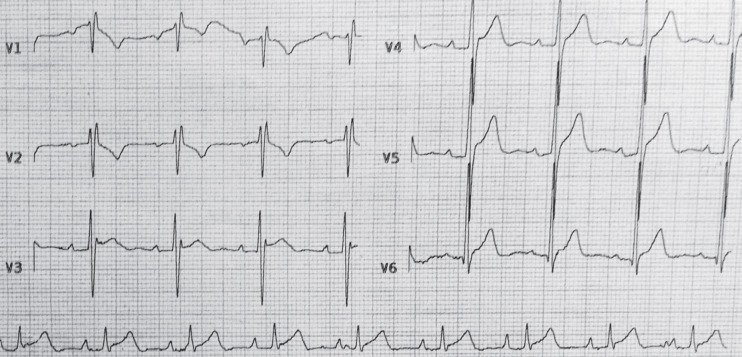
Normal ECG two days after treatment

The patient was referred to the transplant center for better evaluation. Echocardiography showed normal LV and RV sizes and function. The patient underwent coronary angiography and an endomyocardial biopsy was taken. The angiography was normal. Endomyocardial biopsy revealed moderate rejection (ISHLT grade II R). Methylprednisolone (1500 mg) was prescribed for three consecutive days. The palpitation was improved in the second day of therapy. Daily ECG showed a first-degree AV-block with a heart rate of 90 beats/min (Fig 2) after one day of pulse therapy; the rhythm converted to normal sinus (Fig 3) on the following days. A follow-up endomyocardial biopsy taken 2 weeks after the treatment, showed ISHLT grade 0 of rejection.

## DISCUSSION

Bradycardia is a relatively frequent complication in early post-heart-transplantation period. Many of these patients recover spontaneously. Sympathetic denervation, ischemic injury to the sinus node, graft ischemia, and drug effects (e.g., amiodarone) are the common underlying causes of early post-transplantation bradycardia. AV block is relatively uncommon in this period [2].

The exact incidence and cause of AV block after heart transplantation remains uncertain. The available retrospective series are not able to provide an exact prevalence of bradycardia episodes and AV block, but the risk increases over time after transplantation. Complete AV block has been reported late after heart transplantation. Several possible etiologies have been proposed. Those include post-operative injury, progressive conduction system disease associated with coronary artery disease, LV dysfunction, chronic rejection, and injury from endomyocardial biopsies [2]. A potential association of bradycardia with increased likelihood of rejection or graft vasculopathy is controversial [2].

In a review by Anees Thajudeen, bradycardia was associated with acute rejection in a small series, but larger series do not support this observation [2]. There are several reports indicating that late complete heart block or high-grade AV block is associated with rejection and a poor prognosis.

Guanggen Cui, *et al*, report that first-degree AV block is more likely to be related to cellular rejection and coronary artery disease-induced atrial conduction disturbance, whereas second- and third-degree AV blocks are mainly consequences of surgical and catheter intervention injury [3]. Miyamoto, *et al*, reported that 1 of 401 transplant patients developed Mobitz type II AV block 2 years after transplantation. This was associated with an episode of rejection [4].

Avitall, *et al*, show that in the canine transplanted heart, allograft rejection first appears in the right atrium and is much more severe in the atrium than in the ventricle. They propose that the conduction tissue, including the SA node and AV node, are special targets for allograft rejection, and that the right atrial lymphocyte infiltration, myocyte necrosis, and fibrosis associated with acute or chronic rejection might contribute to intra- and inter-atrial conduction disturbances [5]. Cooper, *et al*, reported that 8 of 20 pacemaker implantations late after heart transplantation were associated with episodes of rejection [6].

Carlos Blanch, *et al*, observed a strong relationship between arrhythmia and severe acute or chronic allograft rejection. This association suggests poor prognosis and indicates that these patients should be managed aggressively with close immunological surveillance for cellular and humoral rejection and frequent endomyocardial biopsy and coronary angiography [7].

In conclusion, bradycardia and second-degree AV-block occurring late post-heart-transplantation could be a sign of cardiac allograft rejection. In addition to management of the bradycardia, it is imperative to rule out rejection and treat it immediately.

## CONFLICTS OF INTEREST:

**None declared.**
